# The Psychology Underlying Biased Forecasts of COVID-19 Cases and Deaths in the United States

**DOI:** 10.3389/fpsyg.2020.590594

**Published:** 2020-11-05

**Authors:** Hersh Shefrin

**Affiliations:** Department of Finance, Leavey School of Business, Santa Clara University, Santa Clara, CA, United States

**Keywords:** biases, forecasts, deaths, cases, pandemic (COVID-19)

## Abstract

This paper discusses the impact of a series of psychological phenomena on the U.S. response to COVID-19, focusing on forecasts of cases and deaths. The specific phenomena comprise unrealistic optimism bias, overconfidence, anchoring and adjustment, representativeness, motivated reasoning, and groupthink.

## Introduction

A combination of psychological issues have negatively impacted the manner in which the United States has responded to the COVID-19 pandemic, especially judgments of future cases and deaths.

In mid-September 2020, the number of confirmed cases and the number deaths from COVID-19 in the United States (U.S.) was the second highest in the world. Confirmed cases exceeded 6 million and total deaths exceeded 200,000. On a per capita basis, the U.S ranked second at 19,958 confirmed cases per million and 592 deaths per million, just behind Brazil. By way of contrast, China, the country in which the novel coronavirus (SARS-CoV-2) originated, has experienced just over 90,000 confirmed cases and more than 4,700 deaths, corresponding, respectively, to 62.7 and 3.3 per million.

The situation in the United States is even starker when contrasted with countries such as South Korea [approximately 22,500 confirmed cases (439 per million) and 367 deaths (7 per million)] and Taiwan [500 confirmed cases (21 per million) and 21 deaths (0.294 per million)] which to date rank near the top in best managing the outbreak of the pandemic.

The reasons why confirmed cases and deaths from COVID-19 are so high in the United States are varied and complex. I find it useful to place countries into one of the following four categories:

1. Those that responded aggressively when the virus first presented within their borders, using testing, tracing, social distancing, hygiene, masks, restrictions on mass gatherings, and lockdowns^1^;
2. Those whose first responses were weak, experienced serious outbreaks, and revised their responses along the lines followed by countries who had initially reacted strongly^2^;

3. Those whose first responses were weak, experienced serious outbreaks, and delayed revising their responses along the lines followed by countries who initially reacted strongly, thereby losing control as the virus continued to spread within their borders^3^; and
4. Those who have not yet experienced serious outbreaks^4^.

I suggest that the United States falls into the third category. There are many reasons for the country’s weak response that involve differences in ideology about individual liberties and collective action, regulatory structures, the nature of its public health system, supply chain issues, and flawed human judgment^5^. These are broad issues, and although I will touch on some of these in the paper, I focus mostly on the flawed human judgments made by a small group: the U.S. president, key members of his coronavirus task force, and the Institute for Heath Metrics and Evaluation (IHME) at the University of Washington.

Forecasts by professionals can be important because of their potential to inform the expectations of the public, and to influence the decisions of policy makers. Moreover, there is an important psychological dimension to the manner in which people generally make predictions. In this paper, I discuss one facet of how these issues have been manifest in the U.S. response to COVID-19, by focusing on the presence of optimism bias (Weinstein, 1980) and overconfidence (Svenson, 1981; Harvey, 1997; Hoffrage, 2004) in forecasts of confirmed cases and deaths associated with the pandemic. I also discuss the impact of additional psychological phenomena that contribute to optimism bias and overconfidence, namely motivated reasoning (Kunda, 1990), representativeness (Kahneman and Tversky, 1973), similarity (Tversky, 1977), anchoring and adjustment (Tversky and Kahneman, 1974), and groupthink (Janis, 1972, 1982).

The remainder of this paper is organized as follows. Section “Context: Forecasting U.S. COVID-19 Cases and Deaths” describes the context for the development of projections of cases and deaths from COVID-19 in the U.S. Section “Judgments, Decisions, Biases, and Psychology” focuses on a series of psychological issues that appear to have injected biases into these projections. Section Conclusion concludes.

## Context: Forecasting U.S. COVID-19 Cases and Deaths

On January 28, 2020, U.S. President Donald Trump received a warning about COVID-19 from national security adviser Robert O’Brien, who told him: “This will be the biggest national security threat you face in your presidency. This is going to be the roughest thing you face” (Woodward, 2020). Just over a week later, the president provided an implicit, private conditional estimate of annual U.S. fatalities from COVID-19. The estimate was a range, between 125,000 and 150,000 deaths, conditional on China maintaining control of the virus within its borders^6^. As noted above, total fatalities crossed 200,000 in September 2020.

President Trump’s public pronouncements were diametrically opposed to the views he shared privately with Woodward. In mid-February, the number of coronavirus cases in the U.S. was 15, with all cases having a direct link to China, the source of the outbreak. At that time, the President remarked: “The 15 within a couple of days is going to be down to close to zero” (Watkins et al., 2020).

In the third week of February, the number of confirmed cases began to jump in discrete amounts. U.S. equity market declined sharply, as investors reduced their estimates downwards, of the ability of the U.S. to prevent an outbreak in its homeland (Imbert and Huang, 2020). At the end of February 2020, the number of confirmed cases had risen to 66, with no deaths yet being attributed to COVID-19.

During March 2020, some states within the U.S. began to impose lockdowns and other containment measures to deal with the outbreak of new cases. In consequence, unemployment rose sharply, and both the U.S. Congress and Federal Reserve put anti-cyclical policy measures in place to counteract the negative shock to the economy. At the same time, the messaging from the White House, which had established a coronavirus task force, downplayed the severity of the threat, and emphasized the importance of avoiding unnecessary containment measures that would reduce economic activity. In the third week of March, during a press briefing, the President suggested that the economy might fully reopen by Easter, just a few weeks away^7^.

During March 2020, confirmed cases rose from 69 to 164,620. Total U.S. deaths attributed to COVID-19 rose from 3 to 21,595. In a press briefing on March 29, 2020 (Whitehouse.gov, 2020) the President reversed his views about an Easter reopening, and together with coronavirus task force leaders provided forecast ranges for eventual cumulative U.S. deaths from COVID-19. This particular press briefing was important in three ways.

First, the briefing made clear that the White House accepted that by not engaging in containment measures, total U.S. deaths from COVID-19 would likely be near 2.2 million.

Second, the White House estimated that with containment, total U.S. deaths would likely be between 100,000 and 200,000^8^, although several days later the high end was increased to 240,000 (Bierman and Levey, 2020).

Third, Dr. Deborah Birx, a leading member of the White House task force addressing the pandemic, stated that her team had reviewed the work of 12 institutes that had been forecasting cases and deaths from COVID-19, and pointed people to the IHME’s website, noting that the IHME estimates were in line with their own.

In early April, Eichenbaum et al. (2020) presented a framework that integrated standard models from epidemiology and economics. The paper analyzed the interrelationship among containment policy, economic activity, and the trajectory of cumulative U.S. deaths from COVID-19. The authors examined several cases, and examined a range of outcomes. Their analysis suggested that cumulative U.S. deaths from COVID-19 would be in the range 500,000 to 1.5 million, depending on the strength of containment policy, that herd immunity would be between 50 and 70% of the population, that herd immunity would be achieved between 36 and 52 weeks from the onset of the epidemic, and that full containment of the virus would occur between 75 and 100 weeks after onset. Notably, a weak containment policy would result in herd immunity being achieved more quickly, but with more total cases and deaths^9^.

For the U.S., April 2020 was an important month, and for three reasons. First, confirmed cases and deaths associated with COVID-19 soared and daily rates peaked. At the end of the first week of the month, Dr. Anthony Fauci, arguably the most respected member of the White House coronavirus task force, remarked that the total number of deaths from COVID-19 might not exceed 60,000 (Chappell, 2020)^10^. Third, the White House established its broad strategy for addressing the outbreak. This strategy involved limiting the role of the federal government, delegating responsibility to individual states, providing states with some measure of resources, and working to encourage the weakening of containment measures and consequent reopening of the U.S. economy as quickly as possible. White House personnel working on the response to COVID-19 used the term “state authority handoff” to describe the first part of the strategy^11^.

Although the White House had established a coronavirus task force, within the White House a small group of aides actually separately developed policy for dealing with the virus. This group was headed by the Chief of Staff^12^. Only one member of the group was a public health official, and that was Dr. Birx, an expert in infectious diseases, who had spoken alongside the president at the March 29 press briefing. According to coverage in the *New York Times*^13^, Dr. Birx “was a constant source of upbeat news” and provided “charts emphasizing that outbreaks were gradually easing.” One particular argument she advanced, in April 2020, was that the U.S. “was likely to resemble Italy, where virus cases declined steadily from frightening heights.”

**FIGURE 1:**
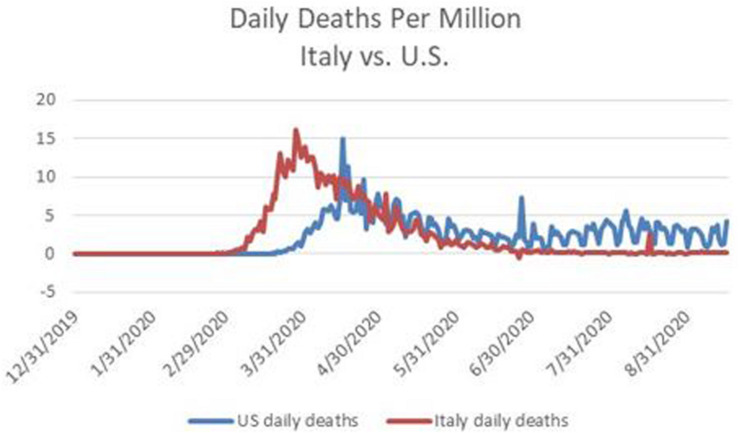
Daily deaths per million from COVID-19 in the U.S. and in Italy between January 1 and September 16, 2020. Source: http://www.ourworldindata.org.

Figure 1 contrasts the number of daily deaths per million from COVID-19 in Italy and in the U.S. between January 1 and September 16, 2020. The left hand portion of Figure 1, from January 1 through the end of April, provides the trajectory relevant for Dr. Birx during March and April.

The perspective provided by Dr. Birx provided support for two White House priorities, namely relaxing containment measures and shifting responsibility for addressing the pandemic to the states. With an upcoming Presidential election in November 2020, the President appears to have been especially concerned that strong containment measures would continue to depress economic activity and therefore the likelihood of his being re-elected.

At the March 29 press briefing, Dr. Birx made clear that the IHME modeling approach, and estimates of cases and deaths, were similar to her own. Subsequently, U.S. media focused attention on the IHME. During April, IHME spokesperson Ali Mokdad, Chief Strategy Officer and Professor of Global Health at the University of Washington, participated in several media interviews to discuss IHME’s projections (forecasts)^14^. On April 15, the IHME indicated that according to their model, the number of new U.S. COVID-19 cases had peaked some days before. At this time, the number of total confirmed cases reached 609,516 and the number of total deaths had reached 26,922.

On April 15, the IHME was projecting that the eventual number of U.S. deaths attributable to COVID-19 would be 60,308. This projection was significantly below the low end of the range provided by the White House, just 2 weeks before, but in line with a statement made by Dr. Fauci a week before. Both the IHME’s statements about the peak daily deaths having been reached, and the lower estimate for total eventual deaths, provided support for those who favored relaxing containment measures and reopening the U.S. economy.

To provide a sense of the economic situation at the time, on April 24, the Congressional Budget Office (CBO) forecast that during the second quarter of 2020 (April–June), U.S. gross domestic product would shrink by approximately 11% from the previous quarter (January–March), which corresponded to an annual rate of 40%^15^. For most of April, the White House had communicated its preference for reopening the economy as soon as possible, and encouraged reopening measures to take place on May 1.

On April 12, at the time of peak daily deaths, IHME Director Dr. Christopher Murray publicly warned that reopening the economy too soon would lead to higher daily deaths^16^. In an interview with *the New York Times*, Dr. Murray noted that on or about April 22, he detected a change in tone in his conversations with Dr. Birx, which reflected a serious interest in reopening the economy imminently^17^. On May 4, when it became clear that the reopening was indeed taking place, the IHME raised its projection for cumulative deaths to 134,475, effectively doubling its prior point forecast.

Infection rates strongly depend on social distancing behaviors. According to coverage in the *New York Times*, the models Dr. Birx employed in her analysis did not properly account for the infection-related implications associated with reopening the economy^18^. Between May 4 and June 19, new daily cases ranged between 18,000 and 28,500, in a series of cycles with no discernable trend. However, thereafter, daily cases began to rise sharply. Writing for the *Washington Post* on June 25, Fritz and Selk (2020) report the highest single-day caseload, over 38,000, for the United States, since the outbreak of the pandemic. Within days, the number of new cases would cross 40,000 (per day)^19^ and during July would exceed 75,000. Fritz and Selk quote Robert Redfield, Director of the Center for Disease Control (CDC) as having said: “Our best estimate right now is that for every case that’s reported, there actually are 10 other infections^20^.”

Fritz and Selk write that according to infectious-disease experts, the increased number of cases reflects a rush to relax containment measures without having put appropriate safety measures in place, which they say “sends a dangerous and inaccurate message”^21^.

During the first week of July, Dr. Birx acknowledged that the U.S. had underestimated community spread of the virus, noting transmission by young people. A month later, she said that the epidemic had entered a new phase, as it had moved into rural areas from urban centers. She was very clear to say that the situation in early August was distinctly different from what it had been during the preceding March and April, in that it had become “extraordinarily widespread^22^.”

During a public presentation in early August, Dr. Birx responded to a question about whether the number of U.S. COVID-19 related deaths would surpass 300,000 by the end of 2020, a figure suggested by a former commissioner of the Food and Drug Administration. Dr. Birx responded to the question by saying “anything is possible,” and noted that such an outcome would be far less likely if Americans practiced appropriate social distancing and avoided mass gatherings (Hawkins and Iati, 2020).

Dr. Fauci regularly emphasized the importance of wearing masks, social distancing, choosing to be outdoors more than indoors whenever possible, avoiding crowds and washing hands. He repeated the point in an exchange with Senator Rand Paul, during an August appearance at a Senate hearing on the nation’s coronavirus response.

Whereas Dr. Fauci argued that these measures just mentioned had helped New York’s recover from a major outbreak in April, Senator Paul held that the recovery reflected herd immunity. Dr. Fauci responded to the herd immunity assertion by stating that 22%, the COVID-19 infection rate in New York, was far too low for herd immunity in the case of COVID-19. However, Senator Paul’s perspective was that other forms of the coronavirus have already provided immunity to the novel coronavirus (SARS-CoV-2), perhaps half the population, in which case the combination would be closer to 70%. By this argument, the U.S. had already reached herd immunity in August, and the pandemic had already begun to wind down in the U.S. (Cook, 2020).

Also in August, the president invited Dr. Scott Atlas into his coronavirus task force and policy group. Dr. Atlas, a radiologist and neuroradiologist and fellow of the Hoover Institution, shared the president’s and Senator Paul’s views about opening the economy, opening schools, and not wearing masks (Cook, 2020). Dr. Atlas’ perspective sharply differed from eminent epidemiologists surveyed by McNeil (2020), whose combined estimates suggest that between 9 and 16% of the U.S. population had been infected by COVID-19. McNeil notes that the top end of this range is much less than the 60% infection rates characterizing areas hard hit by COVID-19, which immunity to coronaviruses other than SARS-CoV-2 did not prevent.

In retrospect, although the U.S. and Europe experienced rapidly rising COVID-19 related cases and deaths during the early months of 2020, by July Europe had managed to reduce new infections and deaths quite dramatically, while the U.S. was experiencing an upsurge (Stancati and Pancevski, 2020). In Europe, new daily confirmed cases peaked at just under 30,000 at the beginning of April, while in the U.S., new daily confirmed cases peaked at just over 30,000 during the second week of April.

**FIGURE 2:**
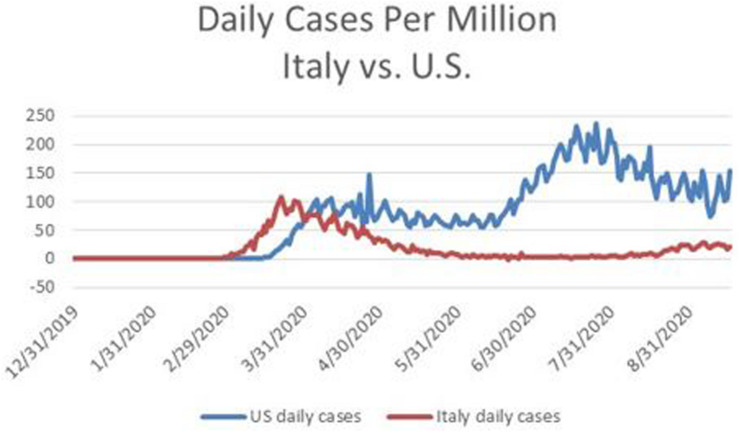
Daily confirmed cases per million of COVID-19 in Italy in the U.S. for the period January 1 through September 16, 2020. Source: http://www.ourworldindata.org.

Subsequently, Europe brought down its daily cases to about 5,000 during mid-July^23^. In contrast, as mentioned above, new daily cases in the U.S. soared above 70,000. See Figure 2 which contrasts the number of daily confirmed cases per million for Italy and the U.S. between January 1 and September 16, 2020. Keep in mind that, as mentioned above, the head of the Center for Disease Control had stated that confirmed cases might severely understate the number of actual infections.

The differences experienced by the U.S. and Europe in July 2020 reflect the different policy decisions made in April 2020; and there is reason to suspect that those policy decisions reflect different judgments about the threat from COVID-19, as well as different preferences about bearing the costs of containment.

Most European governments appeared willing to take responsibility for coordinating a centralized approach, within each country, to testing and tracing, in order to detect and contain emerging clusters of infections. The time series of daily deaths in Italy displayed in Figure 2 reflect the fact that Italy eventually pursued a focused strategy to reduce its new case rate sufficiently before reopening its economy, undertook effective testing and contact tracing, and its population remained vigilant about social distancing.

In contrast, the U.S. followed a decentralized approach that was lacking in coordination. In addition, Europeans appear to be much less concerned about their civil liberties being infringed because of requirements for wearing masks, whereas in some portions of the U.S., required mask wearing was viewed as being highly problematic. In addition, the U.S. has not been able to execute a sufficiently effective strategy for combining testing and contact tracing, which becomes more difficult as the number of cases grows.

## Judgments, Decisions, Biases, and Psychology

I suggest that a series of biases, reflecting the influences of both intentional strategic misrepresentation and unintentional psychological processes, have characterized key judgments and decisions about COVID-19 in the U.S. In this section, I focus on statements, actions, and predictions about the pandemic made by the following key actors: the president, the leading figures in the coronavirus task force, and the IHME. I have organized the section to focus, in turn, on each actor.

The central psychological elements discussed below are unrealistic optimism and overconfidence (in the sense of precision), which have occurred in conjunction with motivated reasoning, elements of groupthink, availability bias, anchoring, and representativeness^24^. I place the psychological issues in bold font, in order to highlight their appearance, and do likewise with strategic misrepresentation.

**The president:** The record is clear in respect to the U.S. president having consistently downplayed the seriousness of COVID-19, and rejected the advice of the scientific community on what would constitute an effective response^25^. In a March 19 call with Woodward (Woodward, 2020), Trump acknowledged: “I wanted to always play it down. I still like playing it down because I don’t want to create a panic.” This statement serves to reconcile the diametrically opposite nature of the president’s public pronouncements about the pandemic, which reflected severely unrealistic optimism bias, and his private views which in retrospect appeared to display only mild optimism bias.

Game theorists use the term strategic misrepresentation to mean actors with agency intentionally disseminating information they know to be untrue, as a means to further their own private interests (Roth, 2002). The record is clear that the president engaged in **strategic misrepresentation**, explaining to Woodward that his motive for making untruthful statements about the pandemic was to avoid creating panic. If so, then to what end?

In an interview with National Public Radio in February, pandemic expert Laurie Garrett suggested that the president’s intent was to downplay the dangers from the pandemic in order to limit damage to the U.S. economy and financial markets, as an economic downturn would threaten the prospects of his being re-elected the following November (National Public Radio, 2020). In May 2020, Garrett stated that the White House was interfering with the CDC, limiting its ability to make pronouncements that reflected the scientific judgments of its staff (Bruni, 2020). The extent of this intimidation became a major media story in September 2020 (Weiland, 2020), and in the first week of October the president, the first lady, and several White House officials tested positive for COVID-19 (Baker and Haberman, 2020).

To summarize the main points about the president’s judgments of U.S. deaths from COVID-19: I suggest that the misrepresentations associated with the president’s public pronouncements on the pandemic largely reflect an attempt to induce bias **unrealistic optimism bias** in a large segment of the U.S. population, including some public officials. In this respect, a key driver of optimism bias is desirability (Weinstein, 1980), interpreted as wishful thinking. I also suggest that **motivated reasoning** has reinforced optimism bias, by inducing this segment of the U.S. public to underweight, if not ignore, the subsequent events of the pandemic that strongly disconfirmed the perspective inherent in the president’s earlier pronouncements. The intent of the misrepresentations, I suggest, has been to foster a political environment that facilitated the relaxation of containment measures at the end of April in order to reopen the economy at that time. As I discuss below, doing so appears to have induced a surge of COVID-19 cases beginning in June and continuing through the summer and beyond, with messaging from the White House that consistently downplayed both the statistics on cases and deaths as well as the views of traditional medical scientists and epidemiologists. That the president himself contracted COVID-19 after flouting the need for masks also appears to be consistent with **unrealistic optimism**.

**IHME:** The IHME uses a proprietary statistical forecasting methodology that makes use of multiple variables. Although the IHME does not provide details of their forecasting methodology, they do say that IHME methodology for projecting deaths is based on models that are different from most other research groups, because of IHME’s emphasis on fitting the patterns of daily mortality observed in the experiences of other geographic areas such as Wuhan, Italy and Spain.

As Dr, Birx stated on March 29, the IHME’s perspective was similar to her own. She also mentioned that she had reviewed 12 different models from institutions that included Imperial College London and Columbia University. Notably, the Reich Lab at the University of Massachusetts, Amherst tracks most of these models, and uses them to compile an aggregate “ensemble” forecast.

**FIGURE 3:**
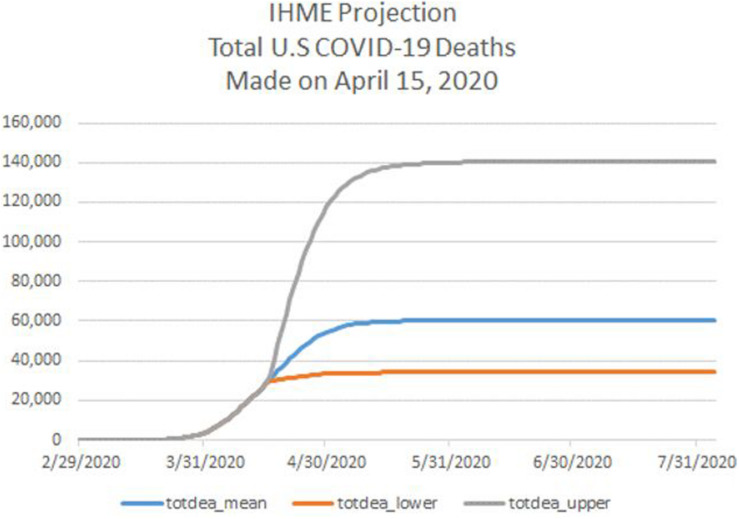
For the period February 29 through July 31, 2020, IHME projection of cumulative deaths from COVID-19 in the U.S., consisting of a point forecast (totdeath_mean) and the lower bound (totdeath_lower) and upper bound (totdeath_upper) of a 95% forecast confidence interval. IHME projection is as of April 15, 2020. Source: www.healthdata.org.

Figure 3 displays the IHME projections, published on April 15, 2020, of the cumulative number of U.S. deaths attributable to COVID-19 for the period April 16 to August 4, 2020. Notice that there are three projections in the figure: a point forecast along with a low forecast and high forecast defining a 95% confidence interval for each forecast date.

According to the point forecast in Figure 3, the COVID-19 outbreak in the U.S. would have been fully contained by August 4, at just over 60,000 deaths, with 95% containment being achieved by May 5. I note that the forecasts of daily deaths, computed as the first difference of the mean cumulative forecast, was the lowest among all professional forecasts of COVID-19 deaths compiled by the Reich Lab, and much lower than the estimates in Eichenbaum et al. (2020). While most forecasts featured positive daily deaths after May 5, the IHME daily forecast fell to near zero after May 5.

Consider whether the forecast(s) displayed in Figure 3 exhibit unrealistic optimism and overconfidence^26^. Formally, unrealistic optimism features the mean forecast of number of deaths being too low, while overconfidence features the width of the confidence intervals being too narrow.

**FIGURE 4:**
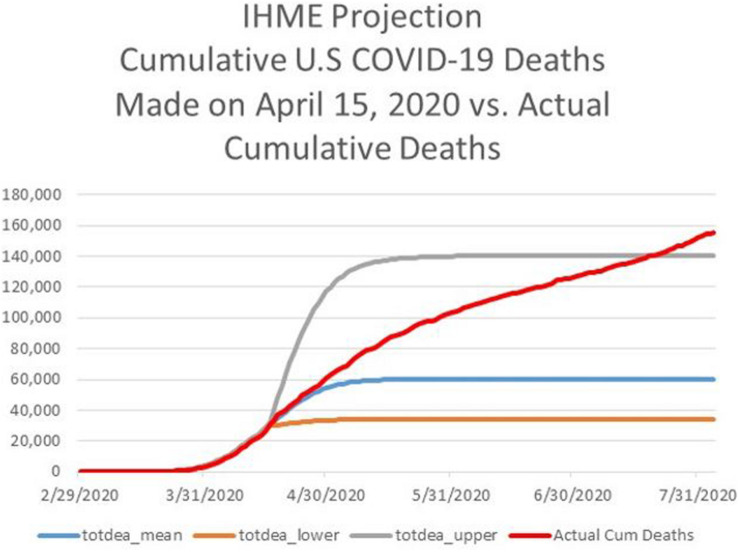
For the period February 29 through August 4, 2020, actual cumulative deaths from COVID-19 in the U.S. (Actual Cum Deaths), IHME projection of cumulative deaths from COVID-19 in the U.S., as of April 15, 2020, consisting of a point forecast (totdeath_mean) and the lower bound (totdeath_lower) and upper bound (totdeath_upper) of a 95% forecast confidence interval. Sources: www.healthdata.org, http://www.ourworldindata.org.

To test formally for **unrealistic optimism**, I compare the IHME mean cumulative death forecast trajectory with the actual death series between April 16 and August 4. See Figure 4, which shows the IHME mean forecast from April 15 lying well below subsequent actual death totals from COVID-19. A formal *t*-test of optimism bias is based on the ratio of the actual series to the point forecast series. With the null hypothesis being no bias, a trend regression of the time series for this ratio should feature an intercept of 1 and a slope coefficient of 0. A trend regression on the actual series exhibits an intercept of 1.0 and a positive slope coefficient with a t-statistic of 96. This result supports the conclusion of unrealistic optimism bias. As can be seen from Figure 4, the IHME projection of full containment by August 4, 2020 also exhibits **unrealistic optimism** bias.

To test formally for **overconfidence** (in the sense of excess precision), I compare the relative frequency with which actual deaths lie outside the IHME’s confidence interval between April 16 and August 4. Overall, the IHME forecast displays slight overconfidence with 85.6%, not the required 95%, of actual deaths lying with the confidence band in Figure 4. However, notice that the IHME’s projections exhibit **underconfidence** in the left portion of the horizon, because the actual series lies completely within the confidence band, and **overconfidence** in the right portion of the horizon, when the actual series moves outside, and remains outside the confidence band.

After the March 29 White House press briefing, American media outlets began to pay disproportionate attention to IHME projections. Professor Mokdar emerged as the chief spokesperson for IHME. As COVID-related deaths surged in the first half of April, Professor Mokdar made clear in interviews that IHME was projecting daily deaths to peak on or about April 12, thereby suggesting that the worst of the pandemic would soon be over. Those views were especially appreciated, and communicated by parties arguing for a rapid reopening of the economy.

I have three points to make about these particular interviews. First, as far as I can tell, media interviews focused only on point estimates, not the wide confidence intervals. Indeed, my impression from viewing several of these videos is that the confidence with which Dr. Mokdar discussed the point forecasts did not reflect the width of the IHME confidence intervals. In this respect, I would characterize the tone of the interview discussions as consistent with **overconfidence** (in the sense of precision).

Second, I note that Professor Mokdar stated in interviews (cited above) that from the first, he and his team have thought that the total number of deaths would not exceed 100,000. Quite possibly, the 100,000 figure served as an anchor, in the sense of **anchoring and adjustment** bias.

Third, Dr. Mokdar did not just confine himself to describing IHME projections, but also offered his opinion on reopening the economy. In this regard, he stated that he thought it was a good time to begin having discussions about reopening the economy in a phased way, and that from the outset IHME had been focusing on both response to the pandemic and recovery. He emphasized the importance of proceeding with a trial approach, so as to prevent the virus from resurfacing after a successful lockdown. He spoke personally about these issues, noting that many of his friends had lost their jobs or had to close their restaurants.

Because of **availability bias**, it is plausible that the media’s attention on IHME led the IHME to exercise disproportionate influence on the views of the American public relative to other information sources. For example, Bierman and Levey (2020) report that based on the IHME projections from early April, cumulative COVID-19 U.S. deaths might even be less than the 100,000 low end forecast which Dr. Birx had communicated in the March 29 White House press briefing^27^.

**FIGURE 5:**
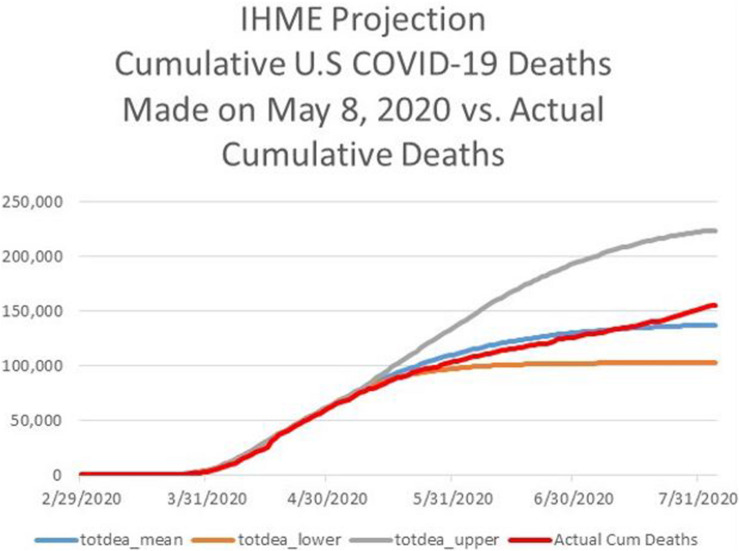
For the period February 29 through August 4, 2020, actual cumulative deaths from COVID-19 in the U.S. (Actual Cum Deaths), IHME projection of cumulative deaths from COVID-19 in the U.S., as of May 8, 2020, consisting of a point forecast (totdeath_mean) and the lower bound (totdeath_lower) and upper bound (totdeath_upper) of a 95% forecast confidence interval. Sources: www.healthdata.org, http://www.ourworldindata.org.

In respect to response, recovery, and biases related to optimism and overconfidence, it is worth noting that on April 12 IHME’s Director Dr. Christopher Murray strongly cautioned that the IHME projections were conditional on not reopening the economy too early; and many states began to reopen at the beginning of May^28^. On May 4, the IHME sharply revised its projections upwards, as displayed in Figure 5. I would also point out that the revised projections were very close to the ensemble forecast produced by the Reich Lab at this time^29^.

The flat portion at the right of Figure 5, meaning the asymptote, for the mean projection in the revised forecast from May 4 was 134,475. Notably, Figure 5 shows that optimism bias disappeared between May 4 and July 4^30^. However, the May 4 forecast was less accurate for the remainder of July as cumulative deaths climbed above 150,000^31^. Even the IHME point forecast of cumulative deaths made on June 27 displayed unrealistic optimism, being more than 4% too low at the end of July with forecasted cumulative deaths not crossing 150,000 until August 8.

During July, the IHME began to make its projections conditional on containment policy. In mid-September, the IHME offered three projections for January 1, 2021: a high forecast corresponding to weak containment (“mandates easing”), a low forecast associated with the universal wearing of masks, and a current projection lying between the low and the high. As of September 18, the point forecasts were, respectively, 445,605, 263,484, and 378,320.

In September, McNeil (2020) reports that the IHME estimated that only 9% of the U.S. population had been infected by COVID-19 at that time, far less than the percentage required for herd immunity. As to the untested theory that immunity from coronaviruses other than SARS-CoV-2 could contribute to herd immunity for COVID-19, McNeil quotes Dr. Murray, the IHME’s head, as saying that this idea is “just nonsense.”

To summarize the main points about the IHME’s projections of U.S. deaths from COVID-19: During April 2020, the IHME’s projections exhibited biases related to both unrealistic optimism and overconfidence. At the time, the forecasts from the IHME were the most closely followed by the U.S. media among all institutions forecasting cases and deaths. I suggest that biased IHME projections during April contributed to fostering a political environment that facilitated the relaxation of containment measures at the end of April in order to reopen the economy at that time. As I mentioned above, doing so appears to have induced a surge of COVID-19 cases beginning in June and continuing through the summer. However, in May, the IHME’s revised projections became less biased in the short-term (up to 2 months out), although continued to exhibit unrealistic optimism and overconfidence in the long-term (beyond 2 months).

**Key members of the coronavirus task force:** During the first week of April, Dr. Fauci publicly stated that the total number of U.S. deaths from COVID-19 might be about 60,000, a figure consistent with the IHME’s point forecast from that period. In retrospect, this was surprising for two reasons. First, it came a week after Dr. Birx had first communicated a lower bound of 100,000. Second, the president’s private estimate for annual deaths was in the range of 125,000–150,000. In any case, just as with the IHME point forecast from that period, 60,000 was much too low, reflecting significant **optimism bias** on the part of Dr. Fauci.

The president’s public pronouncements set the tone for government officials, especially the group within the White House that was charged with setting pandemic policy and which was led by the Chief of Staff. Most of the group members were aides to the President, and only one member was a public health official, and that was Dr. Birx.

Groupthink is a phenomenon in which group members display insufficient devil’s advocacy and are prone to downplay judgmental differences because they feel the need to support the position of the group leader or are concerned that expressing differences of opinion will weaken the group’s esprit de corps. I suggest that elements of **groupthink** operated in White House decision making.

Garrett, quoted in Bruni (2020), speaks of Drs. Birx and Fauci having to “tiptoe around a president’s tender ego.” Coverage by *The New York Times* indicates that during April, Dr. Birx, presented information which supported what the president was hoping to hear, information that would justify reopening the U.S. economy as early as possible. Notably, Dr. Fauci, who was not invited to be a member of the inner group, frequently delivered public messages that were opposite to those of the president, and in July became the target of a campaign by the Chief of Staff to undermine his credibility. In this respect, Dr. Fauci, described himself as “skunk at the garden party” for offering a more pessimistic outlook than what the president had been communicating^32^.

It is possible that there is evidence to the contrary, but if not, it seems plausible to suggest that White House policy makers ignored Dr. Murray’s April 12 warning mentioned above. In this regard, the *New York Times* coverage highlights the failure of Dr. Birx’s framework to control for the impact on reduced social distancing as a result of reopening the economy. In this regard, the IHME reports that social distancing peaked at the same time new (daily) cases, and then began to decline. It is also plausible to suggest that invoking the IHME’s projections when supportive of the policy they favored, but ignoring the warnings when they regarded those warnings as not supportive, is consistent with **motivated reasoning**.

During the first week of August, in a public address, Dr. Birx indicated that the pandemic was entering a new phase in the U.S., as the virus spread into rural areas. Her remarks drew a rebuke from the president, communicated through a tweet. The president suggested that Dr. Birx’s remarks were critical of his policies, and that she was responding to negative remarks about her by the Speaker of the U.S. House of Representatives. The Speaker’s remarks followed the publication of an article by the New York Times (Shear et al., 2020) that contained an unflattering description of Dr. Birx’s role in White House decision making.

The *New York Times* article mentioned that the modeling done by Dr. Birx during April had inappropriately extrapolated the experience of Italy to the U.S^33^. Dr. Birx responded to the article by saying that she wished the *New York Times* would have interviewed her for the article, and emphasized her reliance on data, a practice she had developed in a career spanning four decades.

Being data driven is different from analyzing data using techniques that are unbiased (Fildes et al., 2009; Goodwin, 2017; Harvey, 2007). The issue about placing excessive weight on the experience of Italy when developing predictions about the U.S. relates to psychological biases stemming from reliance on **representativeness** and **similarity** (Kahneman and Tversky, 1973; Tversky, 1977). Did Dr. Birx misjudge the degree to which the U.S. and Italy were similar, and the degree to which the experience of Italy was representative of the situation in which the U.S. found itself? The same questions apply to the projections made by the IHME in April 2020 (see Figures 1, 2).

During a public presentation in early August, Dr. Birx responded to a question about whether the number of U.S. COVID-19 related deaths would surpass 300,000 by the end of 2020, a figure suggested by a former commissioner of the Food and Drug Administration. Dr. Birx responded to the question by saying “anything is possible,” and noted that such an outcome would be far less likely if Americans practiced appropriate social distancing and avoided mass gatherings (see Hawkins and Iati, 2020).

Keep in mind that the IHME projections made in mid-September 2020, and ending January 1, 2021 lie above 300,000 and in addition display no asymptotes, meaning that by January 2021 the IHME’s projection curves had not yet plateaued. Indeed, the IHME website states that IHME leaders believe that the pandemic will be no more than half over by the end of 2020.

In mid-August, Dr. Birx expanded on these points, in remarks at a conference, by coming back to the issue of Italy, saying: “I wish that when we went into lockdown, we looked like Italy. When Italy locked down, I mean, people weren’t allowed out of their houses, they couldn’t come out but once every 2 weeks to buy groceries for 1 hour and they had to have a certificate that said they were allowed. Americans don’t react well to that kind of prohibition” (Mascarenhas et al., 2020). These comments speak to the issues of bias stemming from **representativeness** and **similarity** mentioned above. In respect to the U.S., Dr. Birx also commented that: “Tens of thousands of lives can be saved if we wear masks, and we don’t have parties in our backyards… taking those masks off”^34^.

**FIGURE 6:**
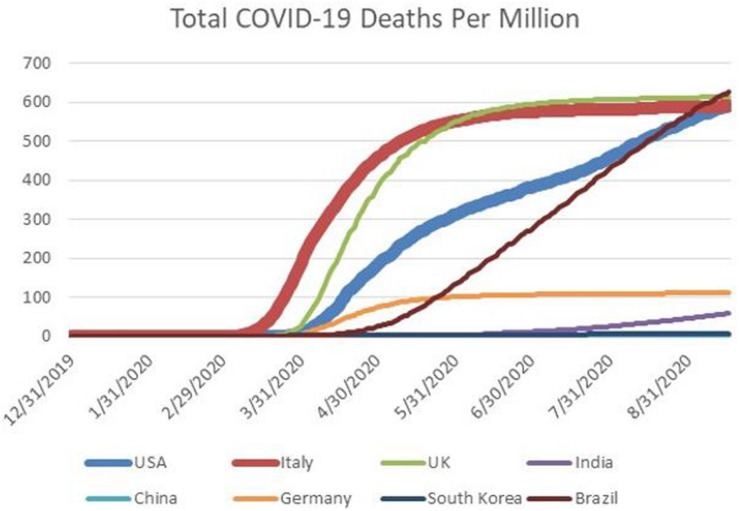
Cumulative deaths from COVID-19, per million, for select countries between January 1 and September 16, 2020. The countries are the United States (USA). Italy, the United Kingdom (UK), India, China, Germany, South Korea, and Brazil. The curves for Italy and the United States are emphasized with thicker lines.

Figure 6 displays the cumulative deaths from COVID-19 for a series of select countries. Notice that the curves for all countries shown, except the U.S. and Brazil reach plateaus at the right. The U.S. and Brazil stand in this regard. According to Dr. Birx, the difference between achieving a plateau, and not, centers on containment policy such as the wearing of masks and social distancing^35^.

To summarize the main points about the judgments of Drs. Birx and Fauci about U.S. deaths from COVID-19: I suggest that unrealistically optimistic forecasts from Drs. Birx and Fauci during April, especially Dr. Birx because of her role in White House decision making, contributed to fostering a political environment that facilitated the relaxation of containment measures at the end of April in order to reopen the economy at that time. As I discussed, doing so appears to have induced a surge of COVID-19 cases beginning in June and continuing through the summer. There is reason to believe that elements associated with groupthink might have impacted Dr. Birx who has struggled to deal with the president’s strategic misrepresentation policy and strong personality.

After negative coverage in July from the *New York Times* about her actions in the White House, Dr. Birx made a series of public statements about weak containment measures in the U.S. Notably, she implicitly explained the source of bias in her April forecasts, namely the over-extrapolation of Italy’s experience with COVID-19. She also downplayed the possibility of reaching 300,000 U.S. deaths from COVID-19 by the end of 2020. However, IHME’s point forecasts from mid-September do indeed feature more than 300,000 U.S. deaths from COVID-19 by January 1, 2021. By the end of 2020, if not before, it will be possible to test whether Dr. Birx’s judgments continued to feature unrealistic optimism bias.

Media reporting indicates that the addition of Dr. Atlas, mentioned above, to the coronavirus task force, has made the work of that body more difficult, or “nightmarish” to use the term attributed to Dr. Birx (Acosta, 2020). Whereas Drs. Birx and Fauci were attempting to emphasize the importance of measures such as mask wearing and social distancing, Dr. Atlas was downplaying the need to do so, while promoting the view that the U.S. was close to or had already reached herd immunity. The president’s public position has been much closer to that of Dr. Atlas who in August began to appear next to the president during press briefings about the pandemic (Acosta, 2020).

## Conclusion

During September 2020, the total number of U.S. COVID-19 deaths surpassed 200,000. This number was considerably larger than the forecasts made in the first 4 months of the year by President Trump, the president’s medical advisers Drs. Deborah Birx and Anthony Fauci, and the Institute for Health Metrics and Evaluation (IHME) at the University of Washington.

The president’s forecasts mostly reflected strategic manipulation, an attempt to induce unrealistic optimism in the U.S. in order to limit containment measures, thereby mitigating the impact on the U.S. economy and financial markets. The manipulation featured a series of psychological phenomena, such as availability bias, desirability, elements of groupthink, anchoring and adjustment, representativeness, and similarity.

Biased forecasts of cases and deaths made by Drs. Birx, Fauci, and the IHME in March and April contributed to fostering a political environment that facilitated the relaxation of containment measures at the end of April in order to reopen the economy at that time. Notably, the IHME’s projections in mid-April were unrealistically optimistic in respect to both total number of U.S. deaths from COVID-19 and the projected dates for full containment. The premature relaxation of containment measures appears to have induced a surge of COVID-19 cases beginning in June that swept across the country.

Drs. Birx, Fauci, and the IHME subsequently revised their April forecasts, stressing the need for the U.S. public to follow prudent containment measures such as wearing masks and maintaining social distancing. The IHME, which in April forecast that by August 4 the pandemic would be fully contained, stated in September that it then expected that on January 1, 2021, the country would only be halfway through the pandemic. Notably, the IHME’s forecasts for more than 2 months out has consistently exhibited overconfidence as well as unrealistic optimism.

Dr. Birx, who had often stood next to the president during his press briefings on the pandemic, and was reluctant to contradict him in public, began to do so in August. Her remarks were especially instructive about some of the thinking in April 2020. At that time her view, and also that of the IHME, was that U.S. fatalities from COVID-19 would follow a similar trajectory as Italy. However, the situation in Italy was not representative of the U.S. in respect to willingness to tolerate strong lockdown measures. Whereas, the government of Italy eventually chose to impose strong lockdown measures, and Italians mostly complied, a large segment of the U.S. population resisted containment, and resonated to President Trump’s messaging on this point. Figures 1, 2, 6 provide a stark graphic visualization of how the experiences of Italy and the U.S. differed.

## Author Contributions

The listed author has approved the article for publication.

## Conflict of Interest

The author declares that the research was conducted in the absence of any commercial or financial relationships that could be construed as a potential conflict of interest.

## Footnotes

1 South Korea and Taiwan fall into the first category. Subsequent infection waves have occurred even among countries falling into the first category.

2 See Stancati and Pancevski (2020). Italy is an example of a country falling into the second category. Other countries that so qualify are China, Germany, Spain, and France.

3 The United Kingdom, Sweden, and Brazil fall into this category, and as I suggest below, so does the United States.

4 Up until the end of July, Botswana and Namibia fell into the fourth category. Since then, confirmed cases and deaths have been increasing.

5 During February 2020, the U.S. Center for Disease Control (CDC) experienced a serious failure in creating a test for COVID-19, which contributed to a major delay in the country’s ability to detect infection (Leonhardt, 2020; Lipton et al., 2020a).

6 In a February 7 taped telephone conversation with Woodward, President Trump said the following: “It’s also more deadly than your—you know, your, even your strenuous flus. You know, people don’t realize, we lose 25,000, 30,000 people a year here. Who would ever think that, right? Pretty amazing. And then I say, well, is that the same thing? This is more deadly. This is 5 per—you know, this is 5 vs. 1% and <1%.” The 125,000–130,000 estimates are inferred from the figures given in this quotation.

7 This focus of this paper is on the narrow issue of forecasting bias, and not the many broad issues relating to problematic judgments and decisions in the U.S. response to the pandemic. Examples of broader issues include President Trump, flanked by members of the coronavirus task force, trumpeting a report that lists the U.S. as being number 1 in respect to the Global Health Security Index, but failing to acknowledge that the report does so for emergency preparedness only, and points out that the U.S. ranks poorly in respect to health care access; the White House in 2018 having disbanded its Pandemic Office, and therefore its readiness to deal with a pandemic; the president firing a deputy inspector general at the Department of Health and Human Services for identifying severe shortages at hospitals that were treating COVID-19; the Center for Disease Control failing to produce a timely test for COVID-19 early on during the pandemic; and the U.S. having a disjointed public health system that was reliant on outdated technology, which limited its ability to conduct testing and contact tracing.

8 The transcript from the press briefing quotes Dr. Birx as saying the following: “So in the model—and there’s a—there’s a large confidence interval, and so it’s anywhere in the model between 80,000 and 160,000, maybe even potentially 200,000 people succumbing to this. That’s with mitigation. In that model, they make full assumption that we continue doing exactly what we’re doing, but even better, in every metro area with a level of intensity. Because we’re hoping that the models are not completely right; that we can do better than what the predictions are.”

9 Value of life models, such as the one used in Eichenbaum et al. (2020) are traditionally used by policy makers can be used to evaluate the tradeoff between COVID-19 infections and deaths on the one hand, and economic activity on the other. Notably, I can find no evidence to suggest that tradeoff frameworks such as Eichenbaum et al. (2020) played any major role, or indeed any role at all, in White House policy decisions related to COVID-19, meaning that policies appear to have been developed based more on intuition than systematic analysis.

10 Chappell (2020).

11 See Shear et al. (2020). In their article, the authors suggest that the state authority handoff strategy would serve to shift blame from the president to the states, in the event that U.S. cases and deaths from COVID-19 surged.

12 The Chief of Staff at the time was Mark Meadows.

13 See Shear et al. (2020).

14 Two examples are a C-Span interview on April 15, https://www.c-span.org/video/?471218-1/tracking-spread-covid-19, and a Fox News April 16 interview on Fox News, https://www.foxnews.com/media/dr-ali-mokdad-ihme-revised-model.

15 This forecast was in line with those made by private economists (Nicholson, 2020). Notably, the CBO also forecast that during the third quarter (July–September), gross domestic product would grow at an annual rate of 23%, in the fourth quarter (October–December) by 13%, and in 2021 (January–December) by 3%. In this regard, the CBO assumed that social distancing will be maintained but at lower levels from those in March and April, through the first half of 2021. The CBO’s forecasts for growth between July 2020 and December 2021 were higher than those of academic economists, the latter having predicted a slower recovery (Baker et al., 2020).

16 CBS interview: https://www.axios.com/imhe-model-coronavirus-social-distancing-93489e69-1e5c-439d-83a6-9d4456d0f52e.html

17 See Shear et al. (2020).

18 See Shear et al. (2020).

19 During March and April, the vast majority of COVID-19 cases and deaths were concentrated in New York State and New Jersey. During the summer, cases and deaths were concentrated in the south and west of the country.

20 For sake of tractability, I do not address the undercount issue, and therefore the analysis I provide can be considered conservative.

21 This comment underscores the relevance of possible optimism bias in professional forecasts of COVID-19 deaths.

22 See Hawkins and Iati (2020).

23 I note that this rate began to increase at the end of July because of reduced social distancing, mostly by young people.

24 References for these issues appear in the introductory section and are not repeated here. In addition, readers are assumed to be familiar with the terminology.

25 See Lipton et al. (2020b).

26 See Shefrin (2020) for a prospective discussion of these IHME projections, rather than a retrospective discussion.

27 For example, according to a Factiva search, for the 3 months ending May 11, IHME projections were mentioned in *The Wall Street Journal* 1,980 times, in contrast to Imperial College London (542 times) and Columbia University (259 times).

28 Before May, the IHME data available on the IHME website did not contain a variable for social distancing. In June, that variable was subsequently displayed. Social distancing, as a proxy for overall strength of containment, is a critical determining variable of the virus transmission rate. Its omission, or underweighting, might well have produced optimism bias in the April forecasts of cumulative U.S. deaths made by IHME.

29 Several of the forecasts monitored by the Reich Lab at the University of Massachusetts have consistently overestimated cumulative U.S. deaths from COVID-19, thereby displaying unrealistic pessimism. The same is true for the framework developed by Eichenbaum et al. (2020), which integrates a macroeconomic model and an epidemiology model, incorporating assumptions about uncertainty in respect to vaccine availability and potential treatments.

30 The revised forecast series actually exhibited mild pessimism.

31 The revised IHME revised forecast from May 10 is more accurate in predicting COVID-19 related deaths during July, ending the month at 146,699, a figure reached on July 26.

32 See Shear et al. (2020).

33 Italy’s initial response to the outbreak was weak, as reflected in the slogan “Milano non si ferma,” meaning Milan does not stop.

34 The same article quotes Jared Kushner, another member of the White House coronavirus decision group, who took issue with Dr. Birx’s comments. Kushner made the point that the group’s intent was to establish policy so that the U.S. healthcare system would not be inundated by COVID-19 patients to the same extent as Italy. In this respect, the number of deaths per capita are similar for the U.S. and Italy; however, Italy only had 25% of the confirmed cases per million as the U.S., and so its fatality rate was much higher. At the same time, cases and deaths per capital have plateaued in Italy relative to the U.S.

35 In the second half of September, corresponding to the right end of Figure 6, new cases began to surge in the UK, France, and Germany.

## References

[B1] Acosta J. (2020). *A ‘Distressed’ Birx Questions How Long She Can Remain on White House Task Force, Sources Say.* Atlanta, GA: CNN.

[B2] Baker P. Haberman M. (2020). *Trump Tests Positive for the Coronavirus.* New York, NY: The New York Times.

[B3] Baker S. Bloom N. Davis S. Terry S. (2020). *COVID-**Induced Economic Uncertainty. National Bureau of Economic Research, Working paper 26983.* Available at: http://www.nber.org/papers/w26983 (accessed April, 2020).

[B4] Bierman N. Levey N. (2020). *New Data Suggest U.S. Coronavirus Death Toll May Not Be As High as Feared.* El Segundo, CA: Los Angeles Times.

[B5] Bruni F. (2020). *She Predicted the Coronavirus. What Does She Foresee Next?.* New York, NY: The New York Times.

[B6] Chappell W. (2020). *Fauci Says U.S. Coronavirus Deaths May Be ‘More Like 60,000’; Antibody Tests On Way.* Washington, DC: National Public Radio.

[B7] Cook N. (2020). *Trump Elevates Scott Atlas, a Doctor With a Rosier Coronavirus Outlook.* Arlington County, VA: Politico.

[B8] Eichenbaum M. Rebelo S. Trabandt M. (2020). *The Macroeconomics of Epidemics. National Bureau of Economic Research, Working Paper 26882.* Available at: http://www.nber.org/papers/w26882 (accessed April, 2020).

[B9] Fildes R. Goodwin P. Lawrence M. Nikolopoulos K. (2009). Effective forecasting and judgmental adjustments. *Inte. J. Forecast.* 25 3–23. 10.1016/j.ijforecast.2008.11.010

[B10] Fritz A. Selk A. (2020). *Coronavirus Updates.* Washington, DC: Washington Post.

[B11] Goodwin P. (2017). *Forewarned: A Sceptics Guide to Prediction.* London: Biteback Publishing.

[B12] Harvey N. (1997). Confidence in judgment. *Trends Cogn. Sci.* 1 78–82. 21223868 10.1016/S1364-6613(97)01014-0

[B13] Harvey N. (2007). Use of heuristics: insights from forecasting research. *Think. Reason.* 13 5–24. 10.1080/13546780600872502

[B14] Hawkins D. Iati M. (2020). *Birx says U.S. Has Entered a ‘New Phase’ of Pandemic as Cases, Deaths Rise.* Washington, DC: The Washington Post.

[B15] Hoffrage U. (2004). “Overconfidence,” in *Cognitive Illusions: A Handbook on Fallacies and Biases in Thinking, Judgement and Memory*, ed. Rüdiger P., (London: Psychology Press).

[B16] Imbert F. Huang E. (2020). Dow Plunges 1,000 Points on Coronavirus Fears, 3.5% Drop is Worst in Two Years. CNBC, February 23, updated February 24. Available online at: https://www.cnbc.com/2020/02/24/us-futures-coronavirus-outbreak.html.

[B17] Janis I. L. (1972). *Victims of Groupthink: A Psychological Study of Foreign-Policy Decisions and Fiascoes.* Boston, MA: Houghton Mifflin.

[B18] Janis I. L. (1982). *Groupthink: Psychological Studies of Policy Decisions and Fiascoes.* Boston, MA: Houghton Mifflin.

[B19] Kahneman D. Tversky A. (1973). On the psychology of prediction. *Psychol. Rev.* 80 237–251.

[B20] Kunda Z. (1990). The case for motivated reasoning. *Psychol. Bull.* 108 480–498. 10.1037/0033-2909.108.3.480 2270237

[B21] Leonhardt D. (2020). *The Unique U.S. Failure to Control the Virus.* New York, NY: The New York Times.

[B22] Lipton E. Goodnough A. Shear M. D. Twohey M. Mandavilli A. Fink S. (2020a). *The C.D.C. Waited Its Entire Existence For this Moment. What Went Wrong?.* New York, NY: The New York Times.

[B23] Lipton E. Sanger D. E. Haberman M. Shear M. D. Mazzetti M. Barnes J. E. (2020b). *He Could Have Seen What Was Coming: Behind Trump’s Failure on the Virus.* New York, NY: The New York Times.

[B24] Mascarenhas L. Yan H. Almasy S. (2020). *Birx Says She Wishes US Lockdown Had Resembled the One in Italy.* Atlanta, GA: CNN.

[B25] McNeil D. Jr. (2020). *Trump Allies Say the Virus Has Almost Run Its Course. ‘Nonsense,’ Experts Say.* New York, NY: The New York Times.

[B26] National Public Radio, (2020). *On the Media, February 28.* Available at: https://www.wnycstudios.org/podcasts/otm/segments. (accessed February 28 2020).

[B27] Nicholson J. (2020). *U.S. Budget Deficit Will Expand to Almost $4 trillion This Year, CBO Says. CBS MarketWatch.* Available at: https://www.marketwatch.com/story/us-budget-deficit-will-expand-to-almost-4-trillion-this-year-cbo-says-2020-04-24. (accessed April 24, 2020).

[B28] Roth A. (2002). The economist as engineer: game theory, experimentation, and computation as tools for design economics. *Econometrica* 70 1341–1378. 10.1111/1468-0262.00335

[B29] Shear M. D. Weiland N. Lipton E. Haberman M. Sanger D. E. (2020). *Inside Trump’s Failure: The Rush to Abandon Leadership Role on the Virus.* New York, NY: New York Times.

[B30] Shefrin H. (2020). *What Makes the COVID-19 Mortality Forecasts Upon Which the White House Relies Seem So Low.* Jersey City, NJ: Forbes.

[B31] Stancati M. Pancevski B. (2020). How Europe kept coronavirus cases low even after reopening. *Wall Street J.*

[B32] Svenson O. (1981). Are we all less risky and more skillful than our fellow drivers? *Acta Psychol.* 47 143–148. 10.1016/0001-6918(81)90005-6

[B33] Tversky A. (1977). Features of similarity. *Psychol. Rev.* 84 327–352. 10.1037/0033-295x.84.4.327

[B34] Tversky A. Kahneman D. (1974). Judgment under uncertainty: heuristics and biases. *Science* 185 1124–1131. 17835457 10.1126/science.185.4157.1124

[B35] Watkins D. Holder J. Glanz J. Cai W. Carey B. White J. (2020). *How the Virus Won.* New York, NY: The New York Times.

[B36] Weiland N. (2020). *Emails Detail Effort to Silence C.D.C. and Question Its Science.* New York, NY: The New York Times.

[B37] Weinstein N. D. (1980). Unrealistic optimism about future life events. *J. Personal. Soc. Psychol.* 39 806–820. 10.1037/0022-3514.39.5.806

[B38] Whitehouse.gov, (2020). *Remarks by President Trump, Vice President Pence, and Members of the Coronavirus Task Force in press Briefing.* Washington, DC: U.S. Government.

[B39] Woodward R. (2020). *Rage.* New York, NY: Simon & Schuster.

